# Relationship between pain intensity, pain catastrophizing, and self-efficacy in patients with frozen shoulder: a cross-sectional study

**DOI:** 10.1186/s13018-021-02693-y

**Published:** 2021-09-01

**Authors:** Junya Hirata, Minori Tomiyama, Yasuhiro Koike, Manabu Yoshimura, Keiko Inoue

**Affiliations:** 1 Faculty of Rehabilitation, Kawasaki University of Medical Welfare, 288, Matsushima, Kurashiki, Okayama, 701-0193 Japan; 2 Hayashi Orthopedic Clinic, 6-1-33 Isegaoka, Fukuyama-shi, Hiroshima-ken, 721-0915 Japan

**Keywords:** Pain intensity, Pain catastrophizing, Self-efficacy, Frozen shoulder

## Abstract

**Background:**

Pain catastrophizing and self-efficacy are useful for predicting pain; these are associated with pain intensity and facilitate evaluation of psychological factors. However, it remains unclear whether the effects are direct or indirect in patients with frozen shoulder; the impact on each variable has also not been clarified. Thus, this study aimed to examine the structural relationship between pain catastrophizing, self-efficacy, and pain intensity in patients with frozen shoulder.

**Methods:**

Participants who were diagnosed with frozen shoulder between January 2016 and March 2017 were recruited from a single orthopedic clinic. Patients aged 18 years or older, who had been symptomatic for < 1 year and reported localized pain in one shoulder, experienced night pain, and had restricted active and passive shoulder motions were included. Pain intensity (Numerical Rating Scale (NRS)), pain catastrophizing (Pain Catastrophizing Scale (PCS)), and self-efficacy (Pain Self-Efficacy Questionnaire (PSEQ)) were measured at the first examination, and the relationship was examined using the Bayesian estimation method. The model was modified repeatedly based on the posterior prediction *p* value, deviance information criterion (DIC), and Bayesian information criterion (BIC); the model with the highest explanatory power was adopted as the final model.

**Results:**

Ninety-three patients diagnosed with frozen shoulder were included in this study. On path analysis, the model in which pain intensity affected psychological factors had the most explanation. The convergence index potential scale reduction was below 1.1, and the convergence of the estimate was confirmed. The posterior prediction *p* value was 0.25, DIC = 1328.705, and BIC = 1356.872; the validity of the fit of the model was confirmed. The path coefficients from the NRS to the PSEQ, from the NRS to the PCS, and from the PSEQ to the PCS scores were − 0.232 (95% confidence interval (CI), − 0.406 to − 0.033), 0.259 (95% CI, 0.083–0.419), and − 0.504 (95% CI, − 0.646 to − 0.334), respectively; these values were statistically significant (*p* < 0.05).

**Conclusion:**

Our results show that pain intensity increases the risk of chronic pain including pain catastrophizing and self-efficacy and that pain catastrophizing increases by decreasing self-efficacy in patients with frozen shoulder.

## Background

Chronic pain negatively impacts activities of daily living (ADL) and health-related quality of life. It has been reported that shoulder pain can be recurrent and frequently progresses to the chronic stage [1]. Frozen shoulder is a condition characterized by a significant decrease of active and passive ranges of motion of the glenohumeral joint along with pain. A previous study with a mean follow-up of 7 years showed that 50% of patients still had mild pain [2]. This suggests that pain relief may promote improved function in patients with frozen shoulder, and appropriate treatment for pain is important. To treat chronic pain, it is critical to control acute pain and prevent the pain from becoming chronic. Melzack and Katz [3] suggested that pain can be interpreted from sensory aspects such as the intensity and location of the pain, emotional aspects (e.g., fear and anxiety), and cognitive aspects (e.g., pain catastrophizing). For example, it has been reported that pain catastrophizing has been associated with increased fear avoidance behaviors, pain intensity, and disability [4]. Pain catastrophizing is the concept of irrational and negative predictions about future events. Sullivan et al. [5] defined pain catastrophizing as an exaggerated negative mental set brought to bear during actual or anticipated painful experience. These results suggest that the cognitive aspects of pain may be as important as the related sensory aspects. Furthermore, Henderson et al. [6] reported that during pain, pain catastrophizing has a significant impact on the activity in motor and sensory integrative regions. In addition, it has been reported that self-efficacy should be considered as a cognitive aspect of pain in the chronic pain model [7]. Bandura [8] defined self-efficacy as people’s beliefs about their capabilities to produce designated levels of performance that exercise influence over events which affect their lives. Self-efficacy related to pain management has been shown to be associated with pain intensity, and it is suggested that doctors and physiotherapists should consider integrating psychological interventions within everyday practice to manage all patients with shoulder pain [9–11]. Although the relationship between psychological factors and pain strength has been examined in patients with frozen shoulder, it remains unclear whether the effects are direct or indirect; the impact on each variable has also not been evaluated.

This study aimed to examine the structural relationship between pain catastrophizing, self-efficacy, and pain intensity in patients with a frozen shoulder. Previous studies [9, 10, 12–16] have reported a correlation between self-efficacy, pain catastrophizing, and pain intensity. Based on these reports, we assumed a path from self-efficacy and pain catastrophizing to the pain intensity, and a path between self-efficacy and pain catastrophizing. Conversely, as pain intensity may affect psychological factors, we assumed a path from pain intensity to self-efficacy and pain catastrophizing.

## Methods

### Ethical considerations

This cross-sectional study was conducted with approval from the local ethics review board (approval number, 16-48). The content and purposes of the study were explained in advance to the patients, and consent to participate in this study was obtained from all the prospective patients. This investigation was conducted according to the principles of the Declaration of Helsinki.

### Patients

Participants who were diagnosed with frozen shoulder between January 2016 and March 2017 were recruited from a single orthopedic clinic at Hiroshima, Japan. Patients who were aged 18 years or older and had been symptomatic for < 1 year and who reported localized pain in one shoulder, experienced night pain, and had restricted active and passive shoulder motions were included. Patients who had calcium deposits or rotator cuff injuries, fractures, work-related injuries or injuries due to accidents, bilateral disorders, and apparent dementia and those who required surgery were excluded. Orthopedic surgeons evaluated the affected shoulder using radiologic examination and magnetic resonance imaging to confirm the diagnosis. The content and purpose of the study were explained in advance to these patients, and consent to participate was obtained from prospective subjects.

### Outcome measures

Pain was evaluated in all patients at the first examination. In order to control measurement bias, the same physical therapists performed all evaluations for each subject. In addition, the questionnaire was solely completed by the patient.

Pain intensity was determined using the Numerical Rating Scale (NRS) [17]. The NRS is a common scale for quantifying pain and is assessed using an 11-point Likert-type scale (0 = no pain; 10 = worst pain). The scale was used to record the average pain intensity in the previous week.

Pain catastrophizing was determined using the Japanese version of the Pain Catastrophizing Scale (PCS) [18], a 13-item self-report questionnaire that measures maladaptive thoughts regarding pain; each item is rated on a 5-point Likert-type scale (0 = not at all; 4 = all the time), and higher scores reflect a greater degree of pain catastrophizing. The PCS contains three dimensions of pain catastrophizing: rumination, helplessness, and magnification. Rumination focuses on pain-related thoughts, helplessness indicates the state of feeling helpless in dealing with a painful situation, and magnification is an overview of the threat of pain; the Japanese version has been found to be valid and reliable [18].

Self-efficacy was assessed using the Japanese version of the Pain Self-Efficacy Questionnaire (PSEQ) [19], a 10-item self-report questionnaire that measures the degree of self-confidence in performing an activity in a painful state. Each item is rated on a 7-point Likert-type scale (0 = not at all confident; 6 = completely confident), and higher scores reflect stronger self-efficacy beliefs; the Japanese version has been found to be valid and reliable [19].

### Statistical analysis

In order to verify the hypothesized model, path analysis was performed by covariance structure analysis. Path analysis can be used to determine selection criteria by measuring the direct influence of one variable on another and by separating the correlation coefficient into direct and indirect effects; additionally, it is possible to analyze multiple variables reflected in the model at the same time [20]. Standardized partial regression coefficients, known as path coefficients, were calculated to allow direct comparison of values; this was performed to reflect the relative importance of independent variables in order to explain the variation in the dependent variable [21]. The Bayesian estimation implemented by the Markov chain Monte Carlo simulation was used for the estimation method, and non-informative prior distribution was adopted. The Bayesian estimation method is an estimation method in structural equation modeling that can estimate the true value or a value close to it even with a complicated model, data that is not normally distributed, or a small size [20]. The reason for adopting the Bayesian estimation method in this study is that there are concerns regarding the stability of data distribution, because the sample size is small. The estimation values are presented as average; the chain number was 5, and the number of simulations ranged from 10,000 to 50,000. The convergence of the model determined that the potential scale reduction would be less than 1.1. The model was modified repeatedly based on the posterior prediction *p* value, deviance information criterion (DIC), and Bayesian information criterion (BIC); the model with the highest explanatory power was adopted as the final model.

Mplus version 8.0 (Muthen & Muthen, http://www.statmodel.com/) was used for statistical analyses; the significance level was set at *p* < 0.05. Individuals with missing values were excluded from the specific outcome measure analysis.

## Results

**Fig. 1:**
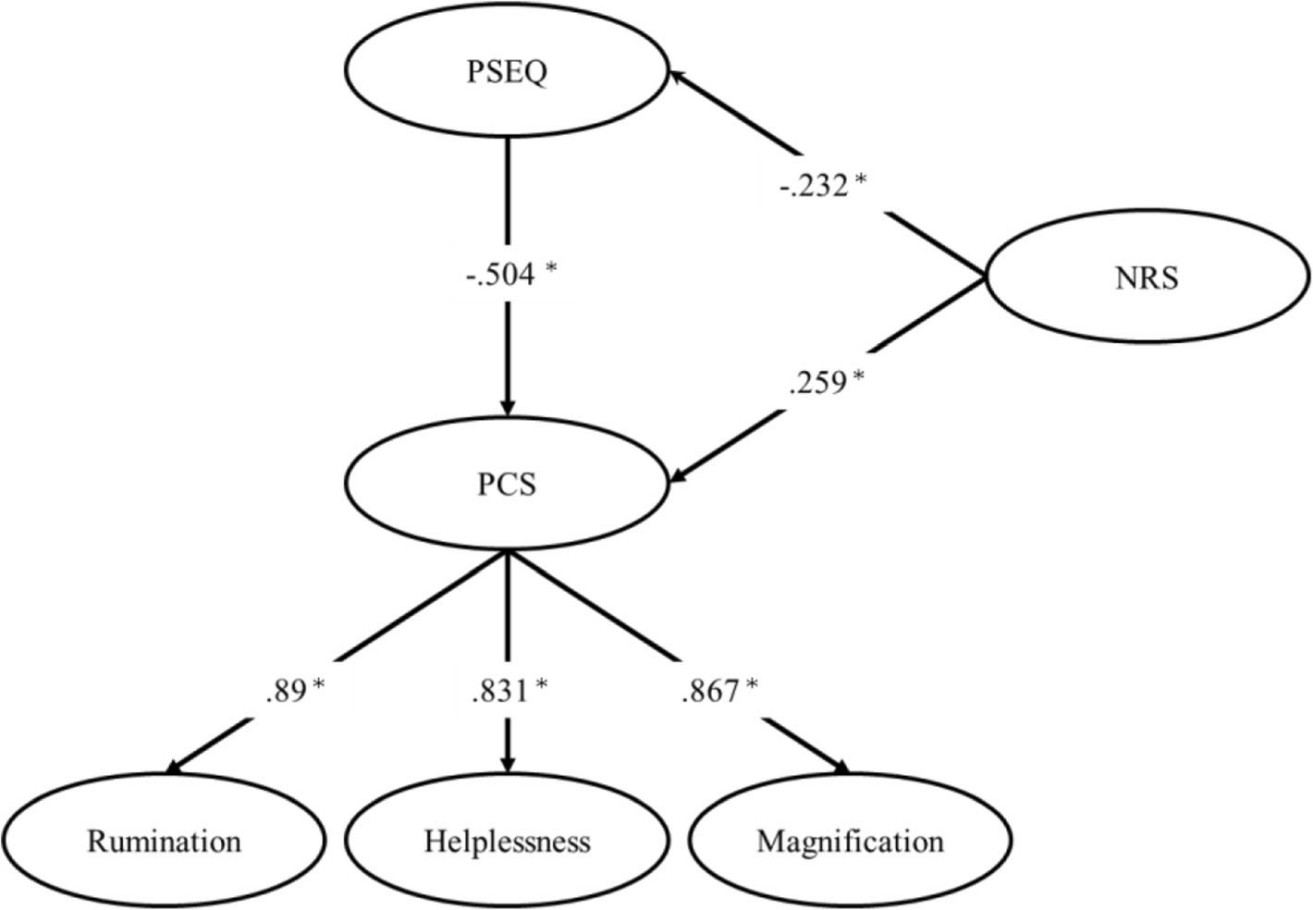
Final model of relationship between the cognitive and sensory aspects of pain. Potential scale reduction < 1.1, posterior prediction *p* value 0.25, DIC = 1328.705, BIC = 1356.872, *p < 0.05. PCS, Pain Catastrophizing Scale; PSEQ, Pain Self-Efficacy Questionnaire; NRS, Numerical Rating Scale

This study enrolled 93 patients; the patient characteristics are shown in Table 1. On path analysis, the model in which pain intensity affected psychological factors had the most explanatory power (Fig. 1). The convergence index potential scale reduction was below 1.1, and the convergence of the estimate was confirmed. The posterior prediction *p* value was 0.25, DIC = 1328.705, and BIC = 1356.872; the validity of the fit of the model was confirmed. Hence, the model shown in Fig. 1 was adopted as the final model. For the factor loading of each factor in PCS, rumination is 0.89 (95% confidence interval (CI), 0.807–0.95), helplessness is 0.831 (95% CI, 0.731–0.899), and magnification is 0.867 (95% CI, 0.776–0.928). These results confirmed that PCS functioned adequately in the subjects of this study. The path coefficients from the NRS to the PSEQ, from the NRS to the PCS, and from the PSEQ to the PCS scores were − 0.232 (95% CI, − 0.406 to − 0.033), 0.259 (95% CI, 0.083–0.419), and − 0.504 (95% CI, − 0.646 to − 0.334), respectively; these values were statistically significant (*p* < 0.05).

**Table 1:** Characteristics and scores for each measurement item of the study patients (*n* = 93)

	Mean (SD)	Range (possible range)
Gender, *n* (%)		
Men	31 (33.3%)	
Women	62 (66.7%)	
Age (years)	61.6 (12.2)	36–85
Disease duration (weeks)	13.5 (15.0)	1–96
NRS	6.4 (2.0)	1–10 (0–10)
PCS		
Total score	22.0 (10.3)	0–47 (0–52)
Rumination	7.3 (3.9)	0–15 (0–20)
Helplessness	10.4 (4.5)	0–18 (0–20)
Magnification	4.5 (2.9)	0–12 (0–12)
PSEQ	44.0 (11.2)	10–60 (0–60)

## Discussion

Previous studies [9, 10, 22, 23] have reported that it is crucial to improve pain catastrophizing and self-efficacy to reduce pain intensity; however, the structural relationship has not been elucidated in patients with frozen shoulder. In this study, we performed path analysis by covariance structure analysis to verify the structural relationship between pain catastrophizing, self-efficacy, and pain intensity in patients with frozen shoulder. The advantage of path analysis is that it allows evaluation of the direction of the influence that could not be assessed by correlation analysis. This study adopted a model in which pain intensity affects self-efficacy and pain catastrophizing and self-efficacy affects pain catastrophizing. The results indicate that pain intensity increases the risk of chronic pain including self-efficacy and pain catastrophizing; it also reduces self-efficacy and increases pain catastrophizing.

The relationship between pain catastrophizing and pain intensity has been studied from various perspectives, including physiological and neurological perspectives. Severeijns et al. [24] proposed an appraisal model of pain catastrophizing, based on the transactional model of stress and coping. It has been considered a problem that pain is not a problem, and the person with high pain catastrophizing experiences the pain excessively and interprets it as not being able to cope. These cognitive schemes enhance pain intensity and pain behavior in order to draw help from others [25]. The fear avoidance model, a cognitive behavioral model of chronic low back pain [26], explains the avoidance of activities that can cause pain because of pain catastrophizing; in addition, becoming sensitive to pain can cause disuse, depression, and further pain. Therefore, it is important to promote behavior change by improving pain catastrophizing. As pain catastrophizing can be an important risk factor for pain in frozen shoulder, our data suggest that it is important not only to reduce pain intensity, but also to increase self-efficacy in order to improve pain catastrophizing.

People with low PSEQ score think that they cannot do anything because of pain. It is important to change the idea that various activities can be performed despite the pain, in order to improve pain catastrophizing. The dimension of generality is assumed in self-efficacy [8]; individuals who think that it is possible to perform movements even if there is pain do not think of pain as a threat, think of pain as something that can be dealt with, and generalize it into pain catastrophizing. In frozen shoulder, the freezing and frozen phases are those in which it may be difficult to cope with and to be active with pain, respectively. These experiences may have reduced self-efficacy and increased pain catastrophizing. Patients with such characteristics and residual pain need to enhance their self-efficacy and reduce pain catastrophizing.

In the model adopted in this study, self-efficacy and pain catastrophizing did not directly affect pain intensity. Mediators such as fear of pain and depression are proposed between pain experience and pain catastrophizing in the fear avoidance model. Therefore, there may be mediators between self-efficacy, pain catastrophizing, and pain intensity in frozen shoulder. Although this study was not able to reveal factors that directly affect pain intensity, the improvement of self-efficacy and pain catastrophizing was not directly considered to improve pain intensity; the influence was considered indirect. Therefore, we believe that when treating pain in patients with frozen shoulder, both psychosocial and biomedical information need to be considered. In particular, in addition to pain control using medications, doctors and physicians may be able to create a virtuous cycle in pain interventions by using approaches to reduce pain catastrophizing.

This study had several limitations. First, path analysis alone is not enough to fully elucidate the causal relationship; this is because the path analysis involves cross-sectional data analysis. In order to address this issue, it is necessary to perform comparisons using multiple hypotheses models and analyze using longitudinal data. Second, the relationship between pain and psychological factors has been studied in a variety of disorders; however, similar results may not be obtained for all conditions generating pain. This study only assessed outcomes for individuals experiencing shoulder pain; hence, the findings may be generalized to other locations with caution. Third, the only relationships investigated in this study were between pain catastrophizing, self-efficacy, and pain intensity. However, various factors have been reported to be related to pain, such as depression and fear [27, 28]. We believe that the relationship of pain will become clearer in the future after examining the factors other than those verified in this study.

## Data Availability

The datasets used and/or analyzed during the current study are available from the corresponding author on reasonable request.

## Abbreviations

ADL: Activities of daily living
NRS: Numerical rating scale
PCS: Pain catastrophizing scale
PSEQ: Pain self-efficacy questionnaire
